# A novel mutation in the *TTN* gene resulted in left ventricular noncompaction: a case report and literature review

**DOI:** 10.1186/s12872-023-03382-w

**Published:** 2023-07-17

**Authors:** Shipeng Tian, Hao Liang, Xiaolei Li, Boce Cao, Lu Feng, Lili Wang

**Affiliations:** 1 Department of Cardiology, Hebei General Hospital, Shijiazhuang, 050000 Hebei China; 2 Hebei Provincial Center for Disease Control and Prevention, Shijiazhuang, 050000 Hebei China; 3 Hebei Medical University, Shijiazhuang, 050000 Hebei China

**Keywords:** *TTN*, Left ventricular noncompaction (LVNC), Case report

## Abstract

**Background:**

Left ventricular noncompaction (LVNC) is a specific type of cardiomyopathy characterized by coarse trabeculae and interspersed trabecular crypts within the ventricles. Clinical presentation varies widely and may be nonsignificant or may present with progressive heart failure, malignant arrhythmias, and multiorgan embolism. The mode of inheritance is highly heterogeneous but is most commonly autosomal dominant. The *TTN* gene encodes titin, which is not only an elastic component of muscle contraction but also mediates multiple signalling pathways in striated muscle cells. In recent years, mutations in the *TTN* gene have been found to be associated with LVNC, but the exact pathogenesis is still not fully clarified.

**Case presentation:**

In this article, we report a case of an adult LVNC patient with a *TTN* gene variant, c.87857G > A (p. Trp29286*), that has not been reported previously. This 43-year-old adult male was hospitalized repeatedly for heart failure. Echocardiography showed reduced myocardial contractility, dilated left ventricle with many prominent trabeculae, and a loose texture of the left ventricular layer of myocardium with crypt-like changes. During the out-of-hospital follow-up, the patient had no significant signs or symptoms of discomfort.

**Conclusion:**

This case report enriches the mutational spectrum of the *TTN* gene in LVNC and provides a basis for genetic counselling and treatment of this patient. Clinicians should improve their understanding of LVNC, focusing on exploring its pathogenesis and genetic characteristics to provide new directions for future diagnosis and treatment.

## Background

Left ventricular noncompaction (LVNC) is a specific type of cardiomyopathy. It is characterized by coarse myocardial trabeculae and deep intertrabecular crypts within the ventricle, which communicate with the ventricular cavity and are associated with systolic and diastolic insufficiency. The gross anatomy of the myocardium is “spongy” and contains two layers, including a shallow dense layer on the surface and a thick, loose, nondense layer inside. LVNC can occur alone or in conjunction with congenital heart disease, genetic syndromes, neuromuscular disease, and other types of cardiomyopathies [1, 2]. Most are found in the apical part of the ventricle, are also seen in both ventricles, and rarely accumulate in the right ventricle alone [3]. This condition was first reported in 1926 in an autopsy with a sponge-like appearance of the myocardium, and the term LVNC was first mentioned in 1990 [4, 5]. The clinical presentation of LVNC is highly heterogeneous and can be asymptomatic. It is detected only on medical examination or through family screening. However, LNVC can also present with end-stage heart failure, malignant arrhythmias, and systemic embolic events. In this context, we discovered a novel mutant of *TTN*.

### Case presentation

A 43-year-old male patient was hospitalized due to chest tightness and shortness of breath that worsened for 5 h. He was previously diagnosed with LVNC and improved after treatment with furosemide, spironolactone, digoxin, amiodarone hydrochloride, metoprolol tartrate, and perindopril tert-butylamine.

He denied a family history of heart disease (his father died, and his mother was alive). Admission examination showed a pulse rate of 100 beats/min and blood pressure of 113/85 mmHg. Lung auscultation shows signs of pulmonary oedema. Heart percussion showed enlargement of the heart border to the left. Additionally, the heart rhythm was definitely irregular, the first heart sound was unequal in strength, the heart sound was low, premature beats could be heard, and no murmur was heard in each valve auscultation area. The rest of the examination did not show any obvious abnormalities.

**Fig. 1:**
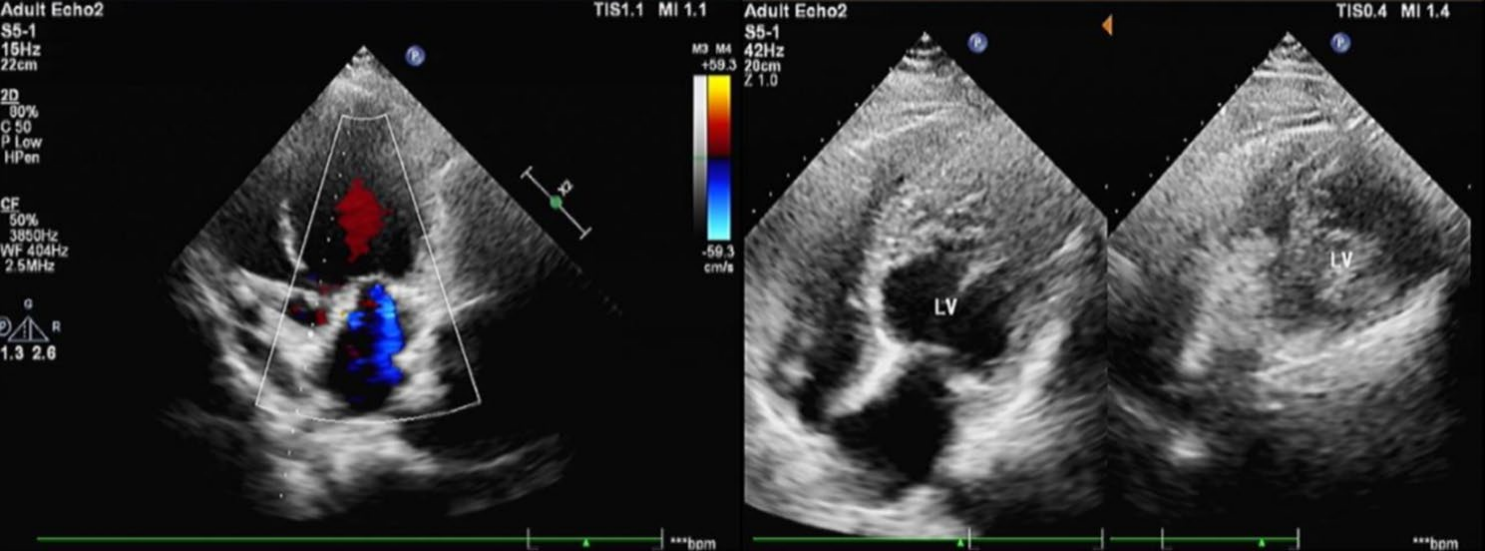
Echocardiography showed increased myofascicular echoes in the posterior lateral wall and apical region of the left ventricle, and the myocardium was loose in texture, forming crypt-like changes

**Fig. 2:**
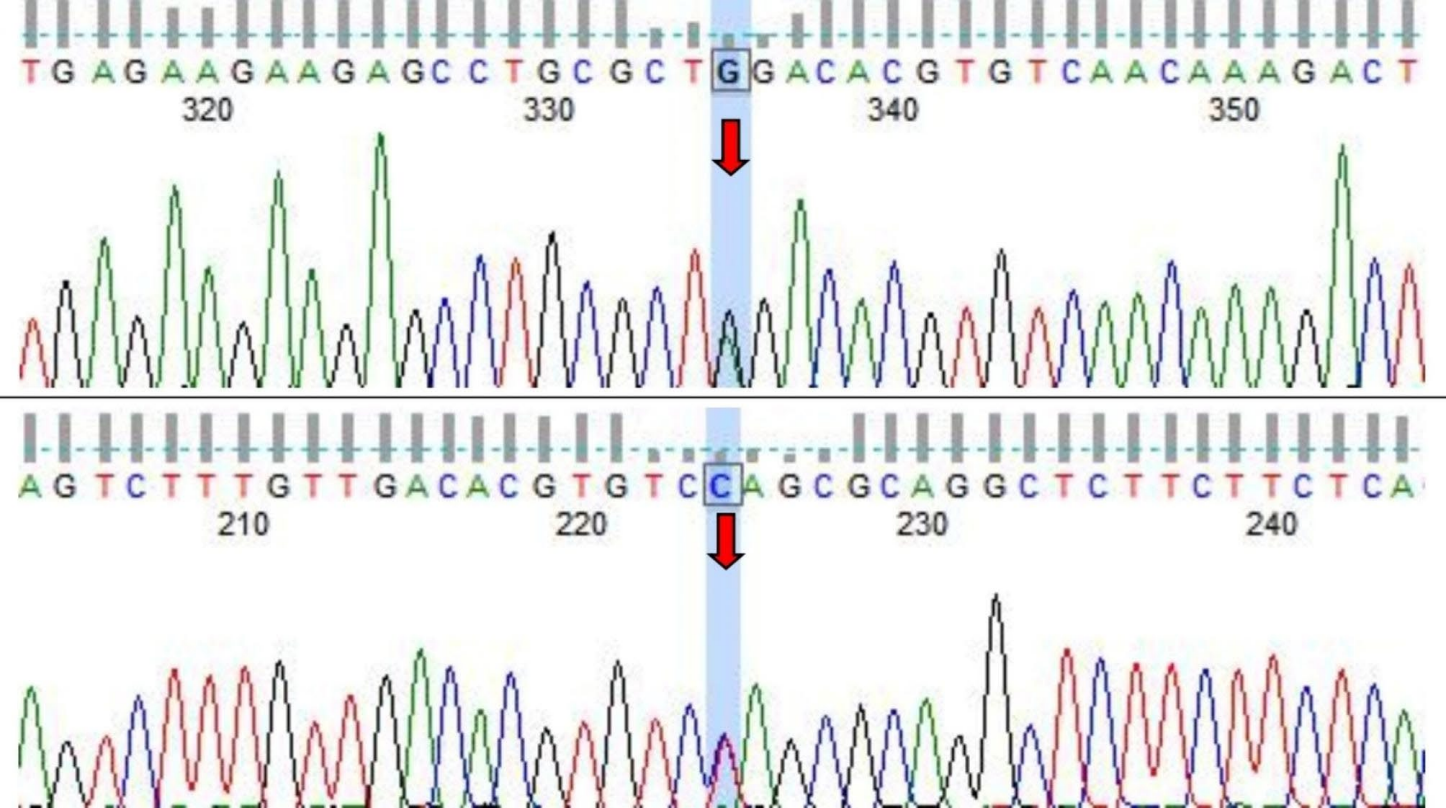
Sanger sequencing validation results of the pathogenic mutation of the *TTN* gene The patient has mutation c.87857G > A in the *TTN* gene (shown by red arrow), resulting in a mutation of the amino acid it encodes to the termination codon (p. Trp29286*)

Bedside chest X-ray shows pulmonary congestion. The echocardiogram suggested LVNC (increased myofascicular echogenicity in the posterior lateral wall and apical region of the left ventricle, myocardial laxity, and crypt-like changes), reduced left ventricular systolic function (left ventricular ejection fraction: 19%, normal range is 50-70%, and left ventricular stenosing rate: 9%), left ventricular dilatation (left ventricular end-systolic internal diameter: 63 mm, and left ventricular end-diastolic internal diameter: 68 mm), trivial aortic regurgitation, moderate mitral regurgitation, mild tricuspid regurgitation, increased pulmonary artery pressure (47 mmHg), and left ventricular diastolic dysfunction (Fig. 1). The electrocardiogram showed atrial fibrillation with tachycardia. The amino-terminal B-type natriuretic peptide precursor was 6559 pg/ml (normal range < 300 pg/ml). Multigene sequencing of the patient for genetic cardiovascular disease revealed a variant in the *TTN* gene, c.87857G > A (p. Trp29286*). This mutation is a nonsense mutation, that can cause the severe loss of function of its encoded protein, titin, and is a heterozygous variant (Fig. 2). The patient was diagnosed with LVNC, heart failure, New York Heart Association (NYHA) Class IV, arrhythmia, and atrial fibrillation. After admission, he was discharged after being treated with rivaroxaban (20 mg, qd) anticoagulation, bumetanide (1 mg, bid) diuretic, sacubitril valsartan and spironolactone (20 mg, qd) to inhibit ventricular remodelling, and amiodarone hydrochloride (0.2 g, qd) to control ventricular rate. The patient was followed up regularly after discharge (after 1 month and 3 months) and was found to have stable symptoms, significant relief of chest tightness and shortness of breath, significant improvement in activity tolerance, and NYHA class I. Echocardiography at 3 months showed LVNC, reduced left ventricular systolic function (left ventricular ejection fraction: 33%, and left ventricular stenosing rate: 16%), left ventricular dilatation (left ventricular end-systolic internal diameter: 56 mm, and left ventricular end-diastolic internal diameter: 67 mm), and increased pulmonary artery pressure (39 mmHg).

## Discussion and conclusion

Currently, morphological diagnosis of LVNC is mainly based on echocardiography or cardiac magnetic resonance imaging (CMRI). Adult echocardiography mainly recommends Jenni’s morphologic criteria. (1) The typical two-layer myocardial structure is divided into a dense epicardial layer and a nondense endocardial layer. The ratio of the thickness of the nondense myocardial layer to the dense myocardial layer at the end of left ventricular contraction in adults is > 2. (2) The main ventricular muscles involved are apical, inferior, and lateral ventricular areas. (3) Color Doppler can detect that the intertrabecular crypts communicates with the left ventricle, but does not communicate with the coronary artery circulation. (4) Excluding the combination of other congenital or acquired heart diseases. CMRI is another method for diagnosing LVNC, mostly uses the diagnostic criteria proposed by Petersen et al. (1) The myocardium is divided into two layers, the dense epicardial layer and the nondense endocardial layer. (2) There are coarse trabeculae and deep intertrabecular crypts in the nondense myocardial layer. (3) Ratio of left ventricular end diastolic nondense myocardial layer to dense myocardial layer > 2.3 [6, 7]. Compared to echocardiography, CMRI is limited by the longer examination time and expensive price. However, it can be used as a complementary tool to LVNC because of the good spatial resolution offered for the whole heart segment [8]. According to current clinical research, patients with dilated cardiomyopathy and hypertrophic cardiomyopathy also often present with hypertrophy and increased, thickening of the myocardial trabeculae, and some scholars believe that the current single morphological diagnostic criteria for LVNC may contribute to the overdiagnosis of the disease in the population [9].

In recent years, with the increasing awareness of LVNC and the advancement of diagnostic imaging techniques, the diagnosis rate of LVNC has gradually increased. However, there are still many controversies about its pathogenesis, and the mainstream theories currently include the embryogenesis hypothesis and molecular genetic mechanism [2, 10]. The early belief was that the failure of the densification process of cardiomyocytes due to abnormal embryonic morphogenesis resulted in the formation of coarse myocardial trabeculae and intertrabecular fossa [11]. In recent years, with the development of molecular genetic research methods, an increasing number of LVNC-related pathogenic genes have been confirmed. These genes mainly include sarcomere protein genes, ion channel genes, and mitochondrial genes, among which sarcomere protein genes are the most common pathogenic genes [12]. Relevant studies have confirmed that the sarcomere protein genes that can cause LVNC include *MYH7, ACTC1, TNNT2, MYBPC3, TPM1, TNNI3*, and *TNN* [13, 14].

The titin gene (*TTN*) belongs to the sarcomere protein genes and contains 364 exons. These exons undergo extensive selective splicing, resulting in many subtypes that range in size from 5604 to 34,350 amino acids, and these subtypes are involved in encoding titin [15]. Titin is a giant sarcomere protein that is widely found in transverse muscle cells and is an important component of sarcomeres. The molecular weight of titin is approximately 35,000 amino acids, and it is known as the third myofilament. It contains four functionally distinct segments: (1) an N-terminus that is anchored at the Z disk; (2) a distensible I band; (3) an inextensible, thick filament binding A band; and (4) a carboxyl M band with a kinase domain. In addition to maintaining the integrity and stability of myocardial fibres, titin also plays a key role in cellular signalling [16]. Previous studies have shown that *TTN* is a common pathogenic gene in dilated cardiomyopathy and hypertrophic cardiomyopathy, and in recent years, the role of *TTN* in LVNC has received increasing attention. In a meta-analysis published by Elham including 2,271 LVNC patients, *TTN* was found to be the most common pathogenic gene in LVNC, with a detection rate of 11% [17].

The molecular mechanism by which *TTN* mutations cause LVNC is unclear, and there are multiple inferences that can be used to interpret it. One possible mechanism is that *TTN* mutations trigger nonsense-mediated mRNA decay (NMD), which detects mRNAs harbouring premature termination, codons, and triggers degradation to prevent the accumulation of truncated and potentially harmful proteins [18, 19]. The Hamedani study found that patients carrying *TTN* mutations had a poorer prognosis and mRNA sequencing indicated significantly lower mRNA expression levels of TTN, indicating that the mutation triggered the NMD mechanism [20]. It is also hypothesized that *TTN* mutations lead to mitochondrial dysfunction, reduce autophagic activity in cardiomyocytes, and aberrantly activates the mTORC1 signalling pathway. Dong further confirmed mitochondrial abnormalities in *TTN* mutant cells, including structural abnormalities observed by electron microscopy, functional abnormalities of reduced extracellular oxygen consumption rate, decreased adenosine triphosphate production and impaired electron transport chain activity. In situ transmission electron microscopy also showed reduced autophagy in mutant cells, accompanied by the activation of the PI3K-Akt-mTORC1 signalling pathway [21].

**Fig. 3:**
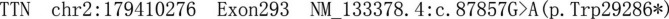
*TTN* gene mutation location *TTN* gene mutation, at chromosome chr2:179410276 position, exon 293, codon 87,857 of NM_133378.4 transcript was mutated from G to A, resulting in a nonsense mutation of amino acid 29,286 encoded from tryptophan to stop codon, a heterozygous mutation

In this case, direct sequencing of the exons of the patient’s gene by second-generation sequencing technology revealed that the *TTN* gene was detected at the chromosome position chr2:179410276, exon 293, codon 87,857 of NM_133378.4 transcript was mutated from G to A, resulting in a nonsense mutation of amino acid 29,286 encoded from tryptophan to stop codon, a heterozygous mutation (Fig. 3), and the American College of Medical Genetics and Genomics (ACMG) Pathogenicity was evaluated as PVS1 + PM2, which is defined as a suspected pathogenic variant [22]. This variant is not currently reported in the literature or the ClinVar, gnomAD and HGMD databases. Neither of the patient’s parents had a family history of heart disease. Combining the patient’s medical history, physical examination, ancillary tests, and genetic testing, the diagnosis of LVNC was made; therefore, the cardiomyopathy developed by the proband was probably due to a novel de novo mutation in *TTN*.

This study identified a novel mutation site in *TTN*, which enriches the spectrum of pathogenic variants in LVNC and provides help for future genetic diagnosis.

## Data Availability

All data generated or analysed during this study are included in this published article.

## Abbreviations

LVNC: Left ventricular noncompaction
NYHA: New York Heart Association
CMRI: Cardiac magnetic resonance imaging
NMD: Nonsense-mediated mRNA decay
ACMG: The American College of Medical Genetics and Genomics
